# Investigation of Critical Geotechnical, Petrological and Mineralogical Parameters for Landslides in Deeply Weathered Dunite Rock (Medellín, Colombia)

**DOI:** 10.3390/ijerph182111141

**Published:** 2021-10-23

**Authors:** Tamara Breuninger, Bettina Menschik, Agnes Demharter, Moritz Gamperl, Kurosch Thuro

**Affiliations:** School of Engineering and Design, Technical University of Munich, 80333 Munich, Germany; sellmeier@tum.de (B.M.); agnes.demharter@tum.de (A.D.); moritz.gamperl@tum.de (M.G.); thuro@tum.de (K.T.)

**Keywords:** dunite, landslide investigation, geological investigation, mineralogical predisposition, pseudokarst, secondary serpentinization, block-in-matrix structure

## Abstract

The current study site of the project Inform@Risk is located at a landslide prone area at the eastern slopes of the city of Medellín, Colombia, which are composed of the deeply weathered Medellín Dunite, an ultramafic Triassic rock. The dunite rock mass can be characterized by small-scale changes, which influence the landslide exposition to a major extent. Due to the main aim of the project, to establish a low-cost landslide early warning system (EWS) in this area, detailed field studies, drillings, laboratory and mineralogical tests were conducted. The results suggest that the dunite rock mass shows a high degree of serpentinization and is heavily weathered up to 50 m depth. The rock is permeated by pseudokarst, which was already found in other regions of this unit. Within the actual project, a hypothesis has for the first time been established, explaining the generation of the pseudokarst features caused by weathering and dissolution processes. These parameters result in a highly inhomogeneous rock mass and nearly no direct correlation of weathering with depth. In addition, the theory of a secondary, weathering serpentinization was established, explaining the solution weathering creating the pseudokarst structures. This contribution aims to emphasize the role of detailed geological data evaluation in the context of hazard analysis as an indispensable data basis for landslide early warning systems.

## 1. Introduction

**Figure 1:**
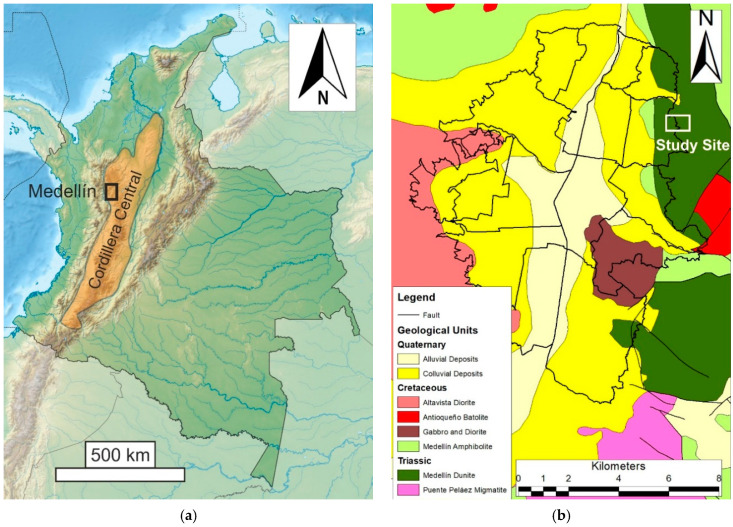
(**a**) Location of Medellín within Colombia and the Cordillera Central; (**b**) The “Comunas” of Medellín (black contours), the location of the study site and the city’s geological units [11].

One of the main Inform@Risk project’s objectives is to concept and install a low-cost landslide early warning system (EWS) in the barrio of Bello Oriente on the eastern border of the city of Medellín, Colombia [1]. The eastern slopes of the Valle de Aburrá are especially prone to landslides, since these are very steep and consist of the Medellín Dunite, a rock highly susceptible to chemical weathering (Figure 1).

Petrology, tectonics and weathering play an important role regarding the geotechnical characteristics and the (temporal and spatial) landslide distribution in the Medellín Dunite. All these factors influence each other and lead to significant small-scale changes. Therefore, every geotechnical intervention in this geological unit needs a detailed exploration of the study site.

Although the Medellín Dunite has been studied by scientists for decades [1,2,3,4,5,6,7,8,9,10], there are still uncertainties regarding its regional characteristics. Its significant small-scale changes in petrology and tectonics are especially a challenge for geologists and hydrogeologists when generating a geological subsurface model.

The Medellín Dunite is part of an ophiolite sequence formed in the Triassic (250–205 Mya b. p.) before the Andean orogenesis in the Pacific Ocean and has already partly been serpentinized by ocean floor metamorphosis [2,10]. Therefore, the Medellín Dunite has already undergone a significant transition. Depending on the study area, the dunite can already be identified as serpentinite [2]. There are regions within the unit that consist of less than 90% (serpentinized) olivine and more than 10% orthopyroxene and hence the rock must be called harzburgite [10]. Other minerals that exist in the unit are amphibole (tremolite, actinolite), talc, chlorite, clinopyroxene, magnesite, mica and serpentine minerals [10]. Due to these deviations from a pure dunite, the unit was renamed several times and has been called Medellín Serpentinite [2], Medellín Dunite Tectonite [3,5], Medellín Dunite [4], Medellín Serpentinized Dunite [6], Medellín Ultramafic Massif [7], Medellín Metadunite [8], Medellín Metaperidotite [9] and Medellín Metaharzburgitic Unit [10]. The term “Medellín Dunite”, however, has prevailed.

Due to the tectonic history, several joint sets can be observed [12]. The first and oldest of those joint sets is the foliation created by ocean floor metamorphosis and the transport to the unit’s current location [2,10]. This foliation (joint set 1) is oriented subhorizontally SW-NE with an incidence angle of max. 30° towards SE [12]. It is characterized by mylonitic structures showing banding of mica, chrysotile and chlorite [12]. The contact to the underlaying Medellín Amphibolite (Rodas Fault) has the same orientation.

The second joint set (joint set 2) strikes NNW-SSE with a subvertical angle of 75–90° towards WSW [12]. This orientation resembles the fault between the Medellín Dunite and the Medellín Amphibolite (La Acuarela Fault), where the amphibolite was uplifted [12].

A third joint set (joint set 3) strikes SW-NE with a dip of 25–60° towards NW [12]. Due to these three main joint sets and several other discontinuities, the whole dunite body is highly disintegrated.

**Figure 2:**
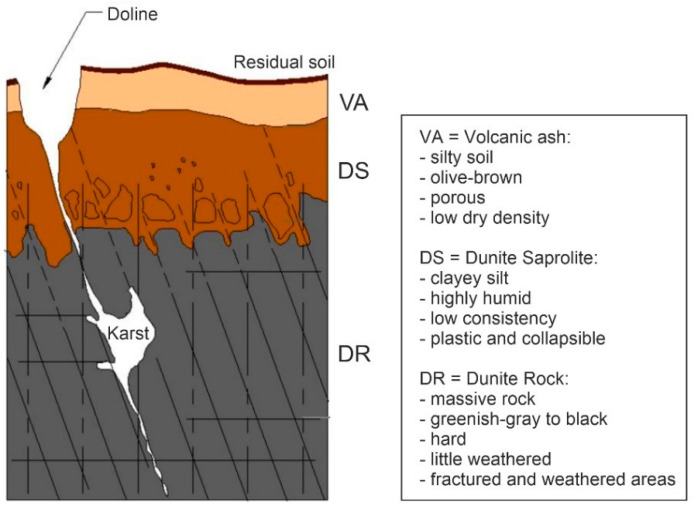
Idealized column profile of the Medellín Dunite with pseudokarst structures; dimensions are not given, because they vary to an evident extent [12] (after Figure 7.6).

Since Medellín is located in a tropical environment, chemical weathering is a strong influencing factor on the subsurface composition. When in contact with water, the rock is altered by degradation into clay minerals and due to oxidation to iron oxides and hydroxides, which weakens the rock’s structure and decreases its compressive strength continuously [13]. The higher the content of olivine in the rock (“pure” dunite), the stronger the weathering [14]. Therefore, the weathering highly depends on the specific petrological composition of the rock and can change within the Medellín Dunite. The typical weathering profile of the unit (Figure 2) consists of organic soil and volcanic ashes on the surface overlaying the saprolite deposits which show a decreasing weathering with increasing depth. This saprolite mostly consists of blocks of at least 50 cm in diameter in a silt-clay matrix with a ratio of approximately 1:1 (block-in-matrix structure). The content of loose material decreases with the depth. The depth of the top of the fractured dunite below the saprolite varies extremely within 0–20 m [12].

This simplified and idealized cross section is influenced by landslide processes taking place along the slope surface (uppermost 10 m), creating a further block-in-matrix structure which occurs to be similar to the one formed by weathering processes (Figure 2) [12].

The phenomenon “pseudokarst” (term here explicitly used as a differentiation from the real karst in carbonate rock) occurs in highly fractured crystalline rock masses. Water circulation in the fractures causes increased weathering and corrosion and, therefore, the solution, and in other places, precipitation of minerals. The Medellín Dunite shows various forms of karst such as dolines, spitzkarren, rundkarren and rillenkarren [12]. The precipitation of the magnesium hydroxide brucite in some fractures [12] is another indication for this phenomenon. Some fractures are filled with clay material, others are open discontinuities of up to 1 m opening width. This pseudokarst phenomenon reaches a depth of at least 60 m [15]. The block-in-matrix structure in combination with the joints opened up by the pseudokarst lead to increased water conductivity which results in an increase of subsurface weathering and pseudokarst formation which again leads to increased water conductivity and subsurface weathering, creating a cycle.

Due to the water absorbent properties of the fine material, the block-in-matrix structure is most likely to be affected by landslides [12] since water infiltration is the main reason and trigger for landslides in the Medellín Dunite. With increasing pore water pressure, the structure “floats” upwards, the friction angle decreases and the slope fails. The blocks tend to “swim” in the matrix and their geotechnical properties do not influence the mechanics of motion once the full detachment occurs [15].

The investigation presented in this article is essential to determine unstable areas that need to be monitored closely by the EWS. It also contributes to the studies on the Medellín Dunite that have already been published and determines whether the findings of previous publications on the subject are also valid in this specific setting.

## 2. Materials and Methods

The underground of the study site was investigated by conducting a combination of field mapping, drillings and laboratory tests. The fieldwork in the project was performed in two campaigns, the first of which took place in August 2019.

During this first field campaign, the landslide features in the study site and several historic landslides were recorded in order to understand the landslide dynamics in this area and to be able to validate numerical models using back analysis methods.

The recording of former events was only possible with the help of the residents, since the whole study site is heavily overgrown with thick grassland and bushland, which makes the registration of morphological landslide indicators difficult. The residents provided information on age, size and speed of several mass movements in the study site [16].

The second field campaign was conducted in February 2020. During this trip, the geological mapping was accomplished. The main goal was to determine the ratio of blocks and matrix in the block-and-matrix structures, since these are most prone to landslides with increasing content of fine material (water storage properties) and the areas with in situ rock, which are not likely to fail. Again, the help of the residents was of great importance.

**Figure 3:**
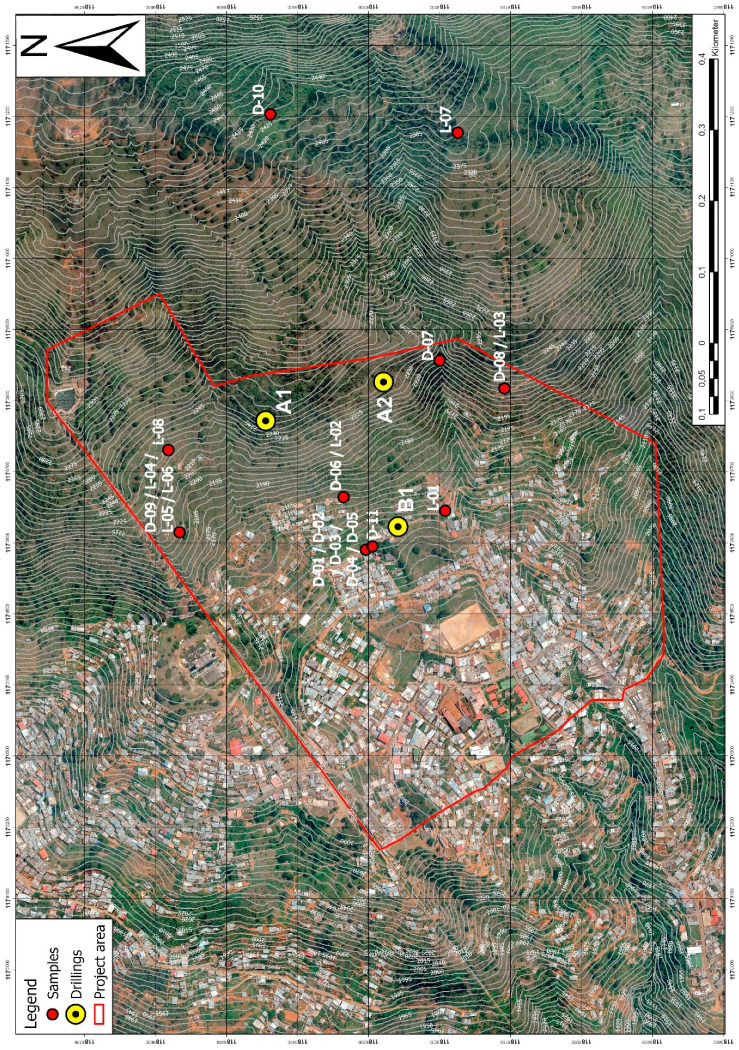
Map of the study site depicting the locations of the drilling and the sample locations (projected coordinate system: MAGNA Medellin Antioquia 2010).

The first drilling campaign was planned for May 2020. Due to the COVID-19-caused lockdown in both Germany and Colombia starting in March 2020, the drillings have been performed in October 2020. The three drilling locations can be seen in Figure 3. All drilling locations were chosen regarding the expected thickness of soil cover (at least 10 m) and their contribution to the early warning system with CSM cables, piezometers and inclinometers being installed after the drilling campaign [17]. A drilling depth of 30.4 m for A1, 30.3 m for A2 and 50.0 m for B1 could be achieved. Drilling B1 is one of the deepest drillings ever performed in the Medellín Dunite [15]. All drillings were performed using the double core rope drilling method without oriented cores. The inner diameter of drilling B1 is 101.6 mm, the inner diameter of the drillings A1 and A2 is 63.5 mm. The bigger diameter in drilling B1 was chosen to fit an inclinometer casing in the borehole in addition to two CSM cables and four piezometers. A1 and A2 do not include an inclinometer casing. All drilling cores were evaluated regarding the core loss, weathering profile, RQD [18], fracture ratio, joint sets and weathering type. Based on all these parameters, six homogeneous areas were established, which are described in Figures S1–S3. A second drilling campaign is planned for the first half of 2021 to complete the picture.

In all the drillings, samples were taken to determine some of the most important soil and rock parameters. The tests that were performed can be seen in Table 1. Due to a limited amount of undisturbed soil material, the soil tests could not be carried out to the intended extent.

Further samples collected from the ground surface during the field investigation in August 2019 were examined mineralogically using X-ray diffraction on soil samples and petrographic thin section microscopy on collected rock samples. The X-ray diffraction was carried out on 8 samples using Cu-Kα radiation and analyzed with graphs created with the software Profex 4.1.0, the microscopy was performed on 11 thin sections from 10 rock samples using a petrographic polarized light microscope. The locations of the sampling are shown in Figure 3.

## 3. Results

### 3.1. Landslide Inventory and Geological Investigation

#### 3.1.1. Landslide Map

**Figure 4:**
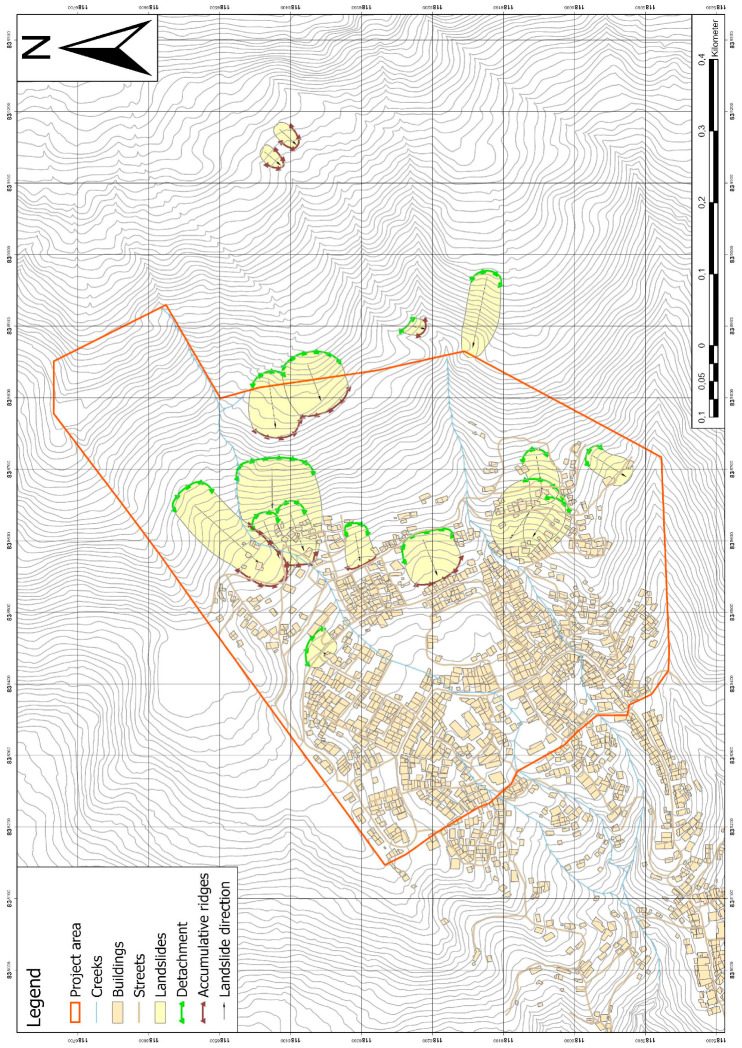
Map of landslide features at the project site, which is marked with the red colored line. The red border represents the project area, the detachment areas are marked in green, the accumulation areas in black. Most of the landslides could be characterized as rotational slides (projected coordinate system: MAGNA Medellin Antioquia 2010).

During the first field trip, an extensive map of the landslide phenomena at the study site was created (Figure 4) on an original scale of 1:3500. The map shows the outlines of former landslide events (the oldest being about 110 years old, the youngest occurred in 2017), their detachment and accumulation areas. Older landslides may be existent but are masked by younger ones.

As a main outcome, it is suggested that most of the landslides are rotational slides, occurring to an increased extent in soil or highly weathered rock [25]; the depths of the landslides can be specified in a range of 5–10 m, which could be classified as shallow to mid-seated [26]. Within the borders of the study area, there are no indications of deep-seated landslides in the past, even though the bedrock is expected to be highly weathered and fractured up to a depth of 60 m or more. However, a deep-seated landslide could not be excluded since the morphological elements would have been eliminated due to anthropogenic influence, such as road, building and plantation construction.

Based on the current results, the probability for a deep-seated landslide in the future is suggested not to be evident, but the possibility cannot be ruled out.

#### 3.1.2. Geological Map

**Figure 5:**
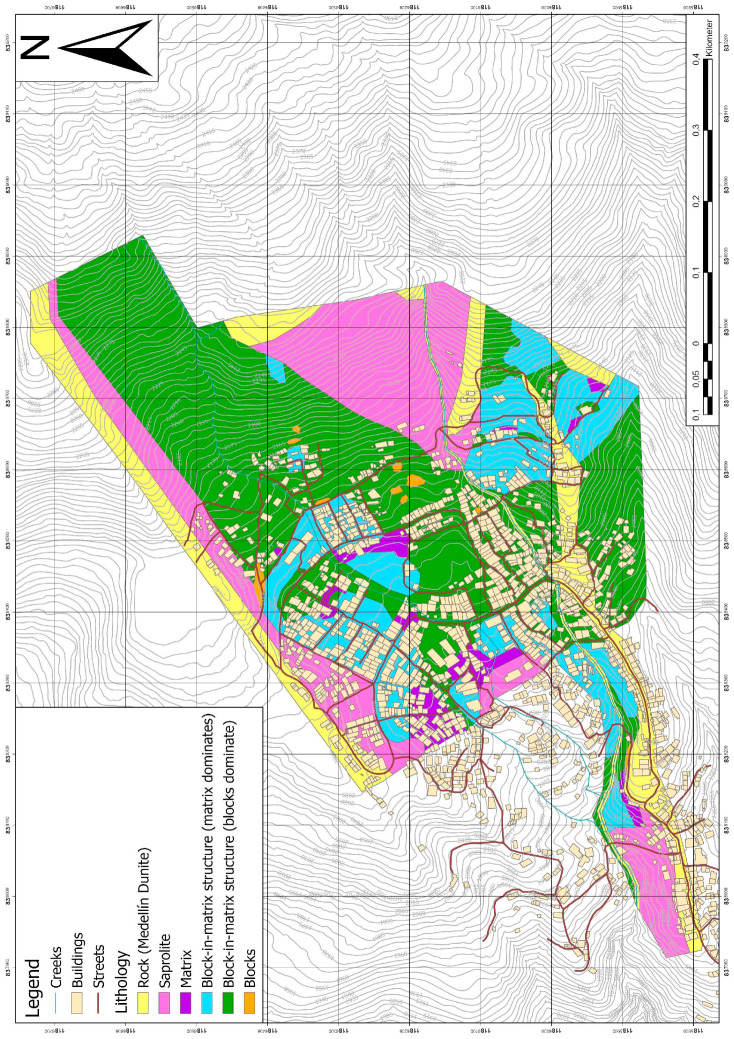
Geological map of the study site. The yellow colors indicate the bedrock ridges mainly in the north and south of the project site (projected coordinate system: MAGNA Medellin Antioquia 2010).

The geological map of the study site is shown in Figure 5 (original scale 1:3500). Two outcrops of in situ rock are ridge structures on the north-western border of the study site and in the south, both structures striking more or less SW-NE. A few other outcrops exist in the eastern part of the site (uphill). All these structures are visible on the surface as ridges, which indicates that these parts of the slope are more resistant and were not moved, e.g., due to landslides, in the past. Another prominent fact is that the strike direction of these ridges could be parallelized with one of the third joint set mentioned in Chapter 1. Therefore, the ridges could have been lifted tectonically creating a horst-and-graben structure in the study site with the trenches being filled by weathering products (block-and-matrix structure) of the described ridges. The in situ rock is heavily weathered and can hardly be distinguished from the saprolite formation.

The saprolite borders the outcrop on the north-western border and also occurs in the mid-eastern part of the study site. Two other outcrops are to the west in the main body of the site and to the far west. The saprolite and the in situ rock are not separated by a sharp line, but by a smooth transition from one to the other. Like the in situ rock, the saprolite has not been moved in the past but is likely to fail in the future.

The block-in-matrix structures that make up the rest of the area (trenches) are subdivided by their block-matrix ratio. Though the main part of the area (green) is dominated by blocks, there are also areas (blue) dominated by matrix. All areas made up of these block-and-matrix structures are very prone to landslides as already discussed in Section 1. A distinction between block-and-matrix structure created by former landslides or created by weathering cannot be made. The origin of the structure, however, does not influence its geotechnical behavior, which is why it is not of immediate importance.

### 3.2. Evaluation of the Drillings

**Figure 6:**
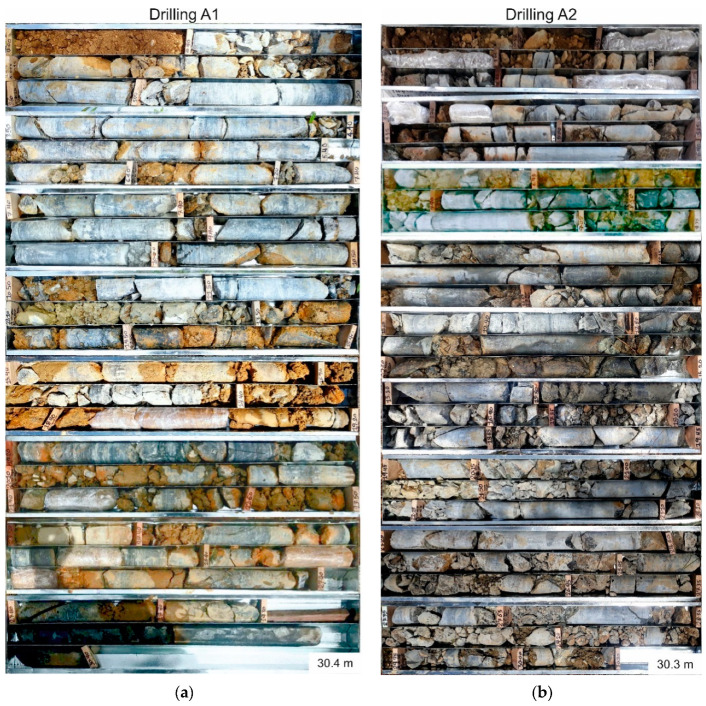
(**a**) Picture of the drilling core of drilling A1; (**b**) Picture of the drilling core of drilling A2. The cores have a length of 1 m each and run from top left to bottom right.

**Figure 7:**
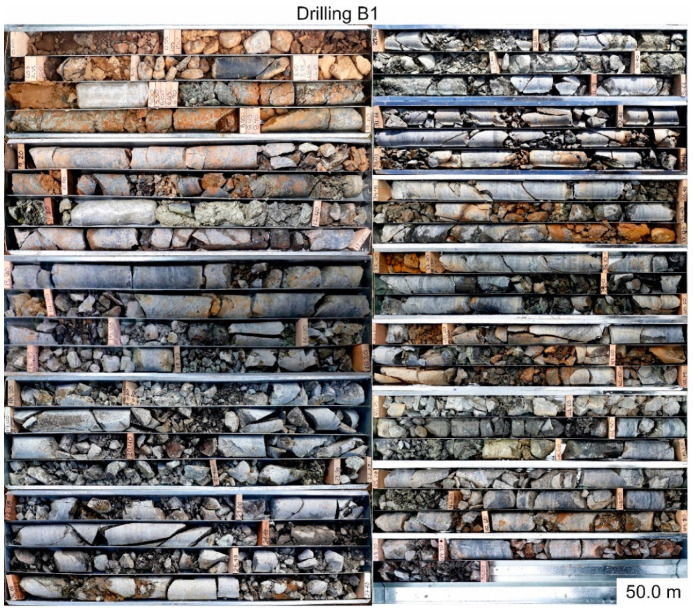
Picture of the drilling core B1, depicted in two rows. The left row shows the drilling core from 0–27.4 m, the right row shows the drilling core from 27.4–50.0 m. The core boxes have a length of 1 m each and run from top left to bottom right.

Figure 6 and Figure 7 show pictures of the drillings cores, Figures S1–S3 include the drilling core documentation (drilling profile) and evaluation with several columns (core loss, weathering profile, RQD, facture ratio, joint set, alteration type and homogeneous area). The evaluation of the drillings was done in collaboration with a Master’s thesis supervised within the project [27].

As depicted in Figure 6 and Figure 7, the thickness of the soil on the surface hardly reaches 2 m, except for drilling B1. In all drillings, there is significant core loss (Figures S1–S3). Except for the first 3.0 m of drilling B1, this core loss is most likely a combination of flushed out, loose material due to the drilling flushing and holes in the ground created by pseudokarst. The first 3 m of drilling B1 are fillings from the road construction; the core loss here is most likely a combination of flushed out loose material and holes due to insufficiently compacted ground.

All three cores show fractures that dip predominantly with 0–15° (foliation/joint set 1) and 30–60° (joint set 3). The few joints dipping >60° are suggested to be parallelized with joint set 2.

Some cores (for the shear and Atterberg limits tests) were wrapped into plastic foil to preserve their original humidity (Figure 6 and Figure 7).

#### 3.2.1. Detailed Description, Drilling A1

In this drilling, the residual soil only reaches 40 cm depth. The material shows a deep brown color and mostly consists of sand, silt and clay. The rest of the core has the best rock quality of all the cores (Figure 6a and Figure S1).

Meters 2.6–12.1 show the weathering stages II after IAEG [28], have an RQD of at least 53 (fairly good) and show joints, which are oriented subhorizontally (most likely foliation/joint set 1) or dip with 30–45° (most likely joint set 3). In this area, some joints show serpentinization and brown colors indicating water circulation through these joints. Other joints show no color changes. These could have been closed prior to drilling due to vertical pressure and, therefore, were not altered by water circulation. The whole area is therefore designated as homogeneous area 2, except for the areas of core loss and meters 0.4–2.6, where the core is more fractured and, therefore, belongs to homogeneous area 3.

Below 12.1 m, the core quality decreases in strength with increased weathering visible due to the brown colors on the core.

The area of meters 12.1–13.5 includes an area of highly destroyed, almost pulverized, but not deep brown colored, loose material. In Colombia, this material is called “salbanda” meaning “fault clay” [15]. It is formed by mechanical grinding without weathering (water circulation). This might indicate a tectonic movement in the past, without an information on the age of this movement. The area has a weathering stage of V and the RQD is 0 (very poor). Therefore, this part of the core is in homogeneous area 5.

The rest of the core (13.5–30.4 m) shows weathering stage III and IV, an RQD of 12–37 (poor to very poor) and mostly contains the joint sets 1 and 3, in meters 13.5–17.4 also one joint of joint set 2, leading to the homogeneous area 4. Brown weathering material dominates the core’s appearance, serpentinization is not or only slightly visible. Two areas in this part of the core show a considerable better quality (19.3–20.1 m and 29.3–30.4 m) with weathering stage II and an RQD of 72 and 75 (fairly good), which results in homogeneous area 2.

#### 3.2.2. Detailed Description, Drilling A2

In general, this drilling core shows less brown fine material compared to the core of drilling A1, but the material is more fractured and fragmented. It also shows more small-scale changes, since the rock quality, weathering and joint numbers change significantly within a few centimeters (Figure 6b and Figure S2).

With up to 2.0 m, the thickness of loose material and soil on the top in drilling A2 is higher than in drilling A1.

Meters 2.0–6.4 show a weathering stage of II to IV, an RQD of 16–60 (very poor to fairly good) and all three joint sets mentioned before (only one fracture of joint set 2). Only one area shows serpentinization, the weathered material mostly shows brown colors. As it is only lightly weathered, this area belongs to homogeneous area 3, except for a small part around 3.5 m which belongs to 4 (in Figure 6b, this part is already wrapped in plastic foil).

The next section of 6.4–7.9 m shows the strongest weathering in the whole core. It has the weathering stage IV with brown and fragmented weathered material, and the ROD is not quantifiable due to the lack of enough rock material. Therefore, this area belongs to homogeneous area 4.

Below 7.9 m, the drilling core shows interchanges of homogeneous area 2 and 3. Homogeneous area 2 is characterized by weathering stage II, an RQD of 36–64 (poor to fairly good), only a few areas with color changes (9.35–11.65 m and 26.95–28.35), indicating a lack of water circulation in the joints and fractures, and mostly joint sets 1 and 3 (joint set 2 only at 15.3–15.8 m). In contrast, homogeneous area 3 shows weathering stages III to IV, an RQD of mostly 0 with spikes of up to 39 (very poor to poor), the weathering types of brown weathered material and fragmentation and the joint sets 1 and 3.

The section 14.3–15.3 m belongs to the homogeneous area 4, because the weathering is much stronger here (weathering stage V).

#### 3.2.3. Detailed Description, Drilling B1

The deepest drilling is also the most complex one (Figure 7 and Figure S3). As the drilling A2, it shows significant small-scale changes and extreme differences regarding the weathering type. All three dominant joint sets are present throughout the core. To a depth of 8.9 m, core loss is the dominating feature. Only 3.05 m of this section are recovered. This phenomenon is most likely caused by flushed out loose material and in the upper 3.0 m additionally by insufficiently compacted ground.

The first 3.0 m of this core are fillings from road construction, mostly gravel and blocks in brown sand and clayey silt.

Meters 3.0–6.9 show weathering stages IV and V and except for one section (3.2–3.4 m/75 (good)), the RQD is not quantifiable. The upper part belongs to homogeneous area 3, since the weathering is still moderate, but the fragmentation is high. The lower part is homogeneous area 4, because of the strong weathering.

In the area of 6.9–11.2 m, the core is still intact but shows extreme weathering (stage V) with brown colors, an RQD of 20 (very poor) and, therefore, belongs to homogenous area 3. This part of the core could be identified as the saprolite overlaying the dunite rock, since the structure of the rock is still visible, but the material is extremely weathered.

The meters 11.2–12.4 are made up of the before mentioned “salbanda” (fault clay); here it appears as gray-greenish silty clay. This indicates movement in the past along this area with no water infiltration, since brown colors are not visible. Due to its crushed structures and clay content, this area belongs to homogenous area 5.

Below 12.4 m, the core shows a variation between homogeneous areas 2 and 3, with some exceptions. Homogeneous area 2 is characterized by the weathering stage II (except for 42.6–44.0 m with IV/V), an RQD of 0–80 (mostly good, but some exceptions) and varying weathering types. Homogenous area 3 shows weathering stages of II–V, an RQD of 0–58 (generally lower than in homogeneous area 2) and more intense weathering colors than in homogeneous area 2.

Some sections in this area show homogeneous area 6. These sections are characterized by weathering stage V, an RQD of 0–50 (mostly under 15) and mostly fragmented cores without brown colors but showing serpentinization. The most prominent characteristic is that the blocks in this area can easily be broken by hand. Therefore, especially the area of 18.2–31.4 m is extremely unstable.

### 3.3. Laboratory Tests

#### 3.3.1. Uniaxial Compressive Strength

**Figure 8:**
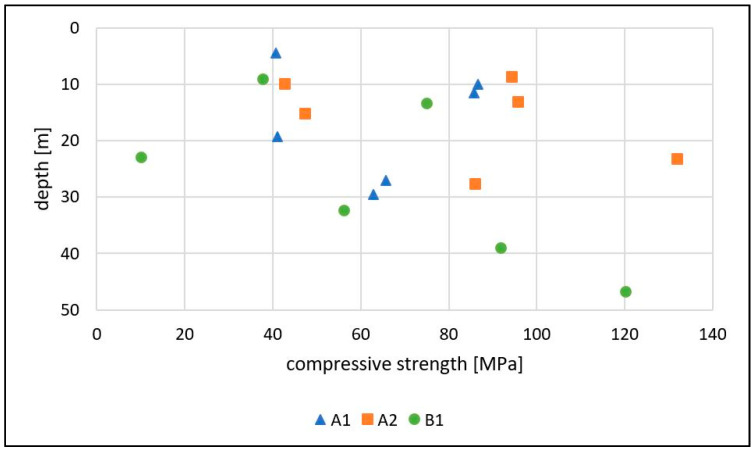
Results of the uniaxial compressive strength tests plotted vs. the sampling depth.

All values of the uniaxial compressive strength are depicted in Figure 8. The compressive strength decreases with increasing weathering [13]. Therefore, it is expected to show lower values with increasing weathering. All samples show weathering stage II on the exterior with little differences.

As visible in Figure 8, there is no correlation between depth and compressive strength in any of the three drillings. The values in general vary from 10 MPa to 132 MPa, which shows the high variation of the materials in the Medellín Dunite.

Most samples (10) have a high compressive strength of 50–100 MPa [29]. One sample has a low compressive strength of 5–25 MPa, five samples have a moderately high compressive strength of 25–50 MPa [29]. Two samples show a very high compressive strength of 100–250 MPa [29].

In drilling B1, the 4 stratigraphically deepest samples show a constant increase of compressive strength with increasing depth, but the two uppermost values do not fit into this correlation. In drillings A1 and A2, only a slight tendency can be seen.

An explanation of this bad correlation might be that the primary rock conditions might be affected by serpentinization and tectonic damage already before weathering. Another one might be that the normal weathering from top to bottom could not be applied in the Medellín Dunite, most likely due to the pseudokarst present in this unit, but also due to landslides, which disturb the uppermost 2–10 m of the unit causing a mix of top layers. Most likely, it is a combination of both.

#### 3.3.2. Brazilian Test

**Figure 9:**
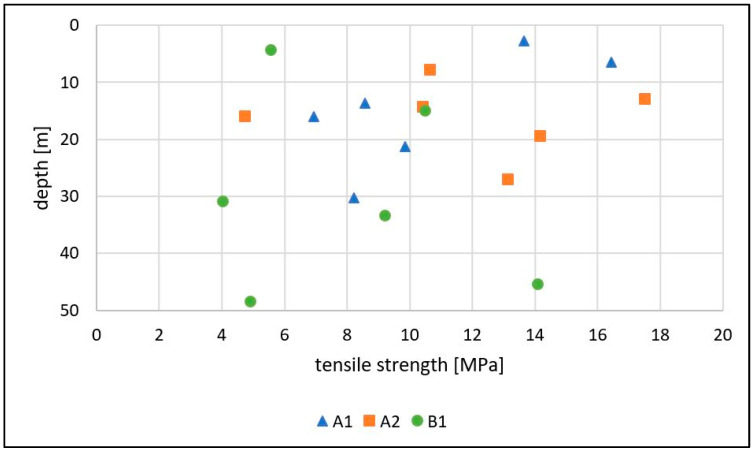
Results of the tensile strength tests (Brazilian tests) plotted vs. the depth of the sampling.

The results of the Brazilian test are depicted in Figure 9. Like the results of the uniaxial compressive strength test, the tensile strength of a sample is expected to decrease with increased weathering [13], but all samples show weathering stage II from the outside with little differences.

Although compressive strength showed at least a slight correlation with depth, tensile strength seems not to increase with the sampling depth at all. This also might correspond to the fact that the primary rock conditions were already affected by serpentinization and tectonic damage before weathering. The values of the tensile strength vary to a high extent between 4.1 and 17.5 MPa.

This was registered in the drilling core analysis, but even the intact rock sections can be highly weathered internally in great depths as the rock tests prove.

#### 3.3.3. Grain Size Distribution

**Figure 10:**
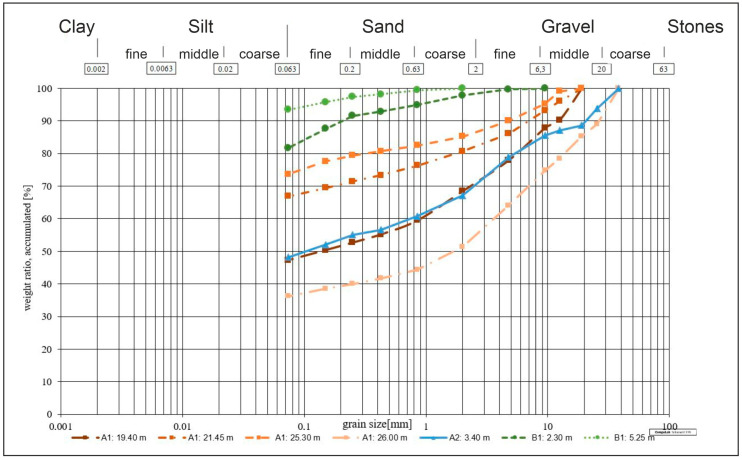
Grain size distribution of all drillings depicted according to DIN EN ISO 14688-1 (2020) [30].

The grain size distribution was determined on seven samples in all three drillings, four of them in drilling A1, one in drilling A2 and two in drilling B1, which are depicted in Figure 10 and Table 2. Even though the tests were performed after INV E 123 (2013) [21], the evaluation was done after DIN EN ISO 14688-1(2020) [30], due to the intercultural collaboration and because ISO standards provide an international standardization level for rock and soil testing and evaluation.

The results of the sieving tests indicate that the loose material in the drilling cores consists of at least 37% silt and clay, in most cases much more. One sample (drilling B1, 5.25 m) even shows an amount of about 93% silt and clay.

Like the rock tests before, this investigation shows no correlation between depth and the values determined. In a weathering profile, the grain size would be expected to increase with increasing depth. This fact could not be observed, which underlines the presence of pseudokarst and disturbance due to landslides.

#### 3.3.4. Atterberg Limits

The results of the Atterberg limits tests are listed in Table 3. All samples could be specified as silt or clay with low to high plasticity after DIN 18196 [31], except for one sample of drilling A1, which has the behavior and grain size distribution of a gravel-silt mixture with a proportion of silt of more than 15% (GU*). After USCS [32], four samples belong to silty or clayey soils (MH and CL), two samples are mixtures of fine material and sand (SC and SM), one sample is a mixture of gravel and silt (GM).

Three samples have a high plastic behavior (TA and UA/SC and MH), indicating a possibly high content of swelling clay minerals. The three samples with moderate and low plastic behavior (UM and TL/SM and CL) could also include swelling clay minerals but to a much lower extent, since their behavior is not dominated by them.

Even though two of the high plastic samples were taken in drilling B1, no clear correlation can be drawn between the place or depth of the sampling and the values.

#### 3.3.5. Direct Shear Test

The drained, consolidated direct shear test was conducted on two samples taken directly from the drilling core with cut-out cylinders in drilling B1 in a depth of 5.0–5.5 m. Therefore, the samples are very similar regarding their geotechnical properties. Unfortunately, it was not possible to gain more than two samples for shear testing due to a lack of soil material in the drillings. Therefore, the results for friction angle and cohesion are only reference values.

Both samples had a diameter of 50.0 mm and a height of 25.4 cm, creating a ratio of approximately 2:1, and were tested with a shear velocity of 0.2 mm/min.

**Figure 11:**
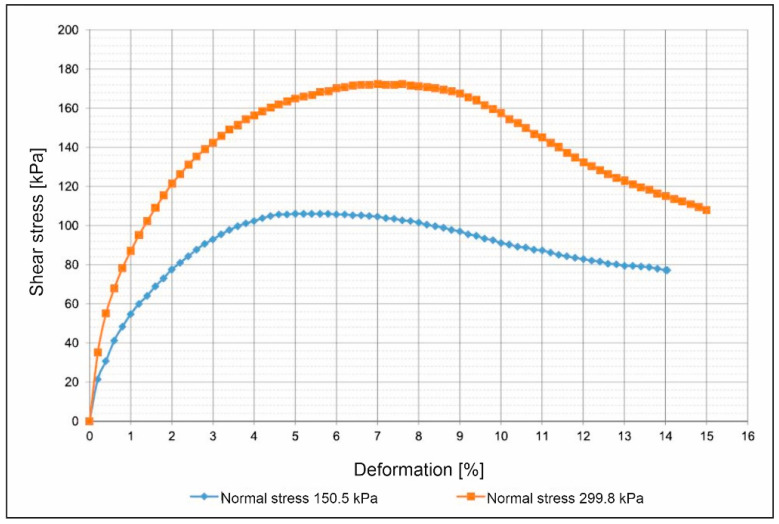
Shear-displacement curves.

**Figure 12:**
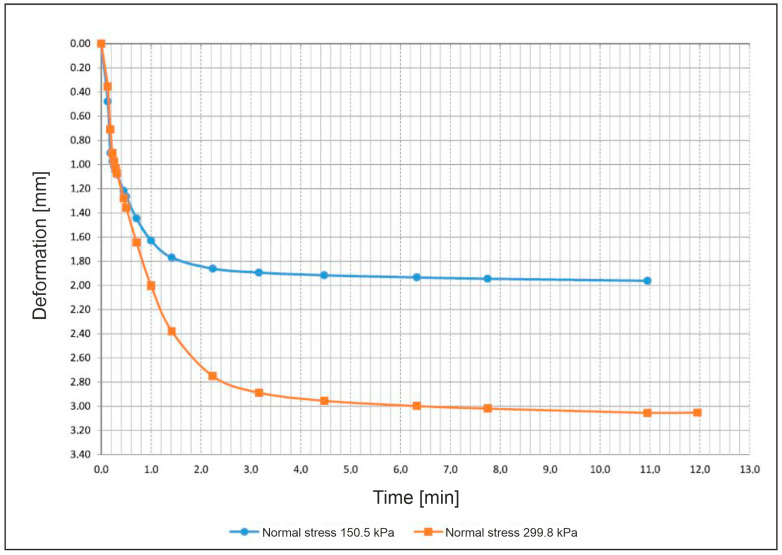
Shear-consolidation curves.

Figure 11 shows the shear-displacement curves: the orange one at a normal stress of 299.8 kPa, corresponding to a load of 588.6 N, the blue one at a normal stress of 150.5 kPa, corresponding to a load of 295.5 N. The form of the curves indicates that the soil is densely packed since the curves drop after reaching their maximum shear stress at the point of failure. In addition to the shear-displacement curves, the shear-consolidation curves (Figure 12) show a consolidation of the samples during the shear test, indicating a compaction of the soil. This shows that despite the dense compaction shown in Figure 11, the soil is not over-consolidated and can still be further compacted.

The point of failure of the sample sheared at a normal stress of 299.8 kPa is 172.4 kPa and the failure point of the sample sheared at a normal stress of 150.5 kPa is 106.1 kPa, resulting in an effective friction angle φ of 24.0° and a cohesion *c* of 39.2 kPa.

A friction angle of 10–25° and a cohesion of >10 kPa are characteristic for strongly cohesive soils [33]. This behavior of the loose material in the drilling cores is already apparent from the results of the sieving tests and the Atterberg limits tests, which mostly identified fine material with plastic behavior. Since the samples were disturbed by the drillings process, the results have to be compared with those of undisturbed samples taken from the surface.

### 3.4. Mineralogical Investigations

#### 3.4.1. Petrographic Thin Section Microscopy

**Figure 13:**
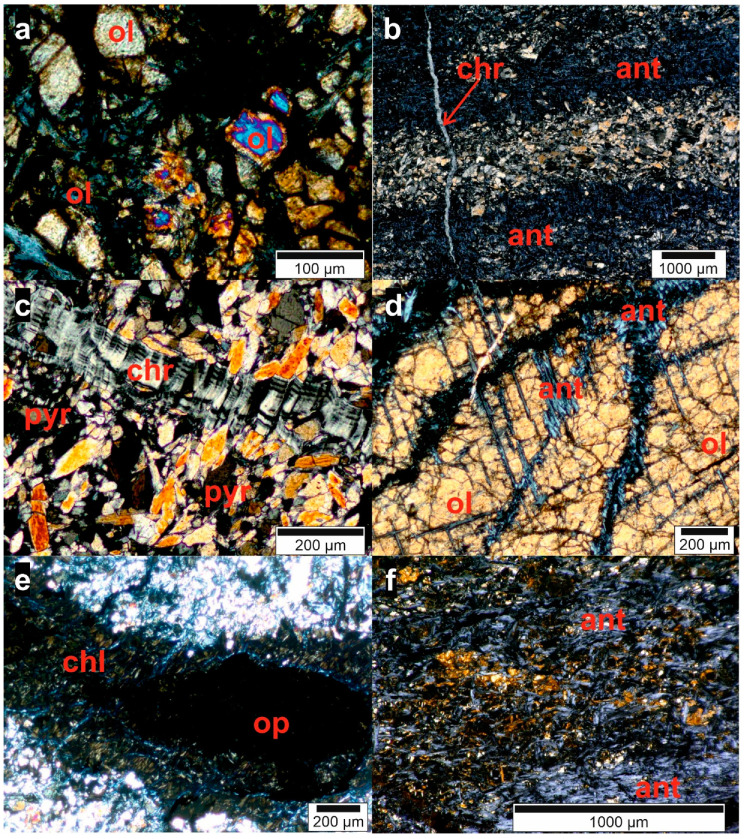
Microscopic photos of the thin sections related to the sample numbers: (**a**) = D-01; (**b**) = D-02; (**c**) = D-03; (**d**) = D-04; (**e**) = D-05; (**f**) = D-06 (ol = olivine, ant = antigorite, chr = chrysotile, pyr = pyroxene, amp = amphibole, chl = chlorite, op = opaque phase) [34].

**Figure 14:**
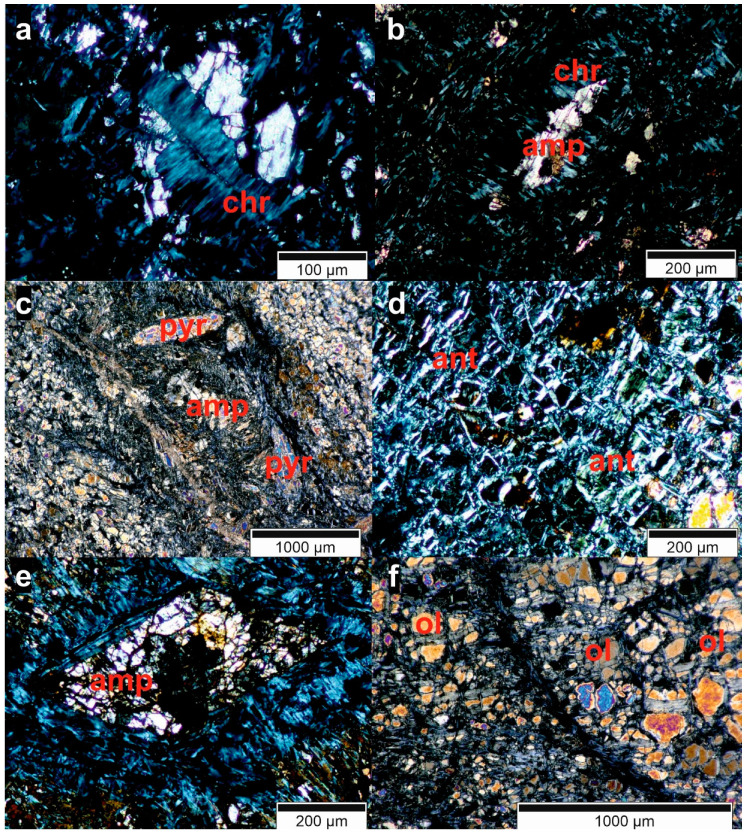
Microscopic photos of the thin sections related to the sample numbers: (**a**) + (**b**) = D-07; (**c**) = D-08.1; (**d**) = D-08.2; (**e**) = D-09; (**f**) = D-10 (ol = olivine, ant = antigorite, chr = chrysotile, pyr = pyroxene, amp = amphibole, chl = chlorite, op = opaque phase) [34].

For the petrographic analysis, a Bachelor’s thesis [34] has been supervised and the 11 thin sections were analyzed in collaboration. During this analysis, six minerals or mineral groups could be identified frequently (Table 4). There are also other minerals in the samples, but in very small frequency and quantity. In Figure 13 and Figure 14, some microscopic pictures of the thin sections show typical shapes of the minerals in the rocks and their weathering, which is visible in some samples.

Since dunite consists of olivine and pyroxene, the presence of these two minerals has been expected. The serpentinization is very advanced in most samples, as is the weathering which is creating chlorite. The opaque phases could be ferrous minerals such as hematite and magnetite, which are common in ultramafic rocks.

The olivine crystals in the samples are mostly broken and heavily weathered at their exterior, as is visible in Figure 13a,d and Figure 14f.

Pyroxene (Figure 13c and Figure 14c) is not present in all samples, suggesting these parts of the rock might either be dunite in a strict sense without any pyroxene or that the pyroxene minerals have already been altered by serpentinization or weathering.

Five samples contain amphibole (Figure 14c,e), which is commonly present in ultramafic rocks. Its absence in the other thin sections indicates that it is already serpentinized.

Figure 14b shows serpentinization of an amphibole from the outside. Serpentine was found in most samples, either chrysotile (fiber serpentine, Figure 13b,c and Figure 14a,b) or antigorite (foil serpentine, Figure 13b,f and Figure 14d). The serpentinization starts at fractures (Figure 13d and Figure 14a) and grows into the minerals from there.

Chlorite (Figure 13e) is found in all samples. It is a common mineral in metamorphized ultramafic rocks and, therefore, was expected to appear in the Medellín Dunite [35]. It is also a weathering product of silicates such as olivine [35].

Sample D-07 also contains several quartz veins. These veins are created by precipitation of SiO_2_ in fractures. The SiO_2_ is dissolved in water circulating in the fractures, its origin lies in other silicate minerals in the rock (olivine, pyroxene, amphibole, etc.).

Samples D-01, D-04, D-05, D-06 and D-09 contain small amounts of goethite. Goethite is a FeO(OH) mineral formed by weathering of iron containing minerals like pyroxene, amphibole and olivine and, therefore, its presence in the Medellín Dunite is to be expected.

The differences in the mineral content show that the Medellín Dunite is not homogeneous regarding its mineralogical composition. Serpentine, olivine and chlorite seem very common, but other samples were free of one of those minerals. Additionally, the domination of serpentine in the rock suggests that the dunite has already been transformed into a serpentinite.

Since all samples were taken from the surface without knowledge of their origin in the slope but as typical representative specimens of the rock material, there is no possibility of correlating the results with the sampling location.

#### 3.4.2. X-ray Diffraction Analysis

The analysis of the X-ray diffraction was conducted in collaboration with a Bachelor’s thesis [36] supervised within the project. The mineral content of the 8 analyzed samples is depicted in Table 5 [36]. All samples contain chlorite, amphibole (tremolite), quartz and hematite. Except for sample L-07, goethite is present in all samples; serpentine (lizardite) is missing only in samples L-05 and L-07. Other minerals that were found in some samples are gibbsite, magnetite and olivine (forsterite).

All minerals found in the samples are to be expected in weathered material of ultramafic rocks. The quartz content seems uncharacteristic at first sight. Quartz, however, is very resistant to weathering and, therefore, even a very small amount of it (veins in the rock, see Section 3.4.1) is enriched very fast in its weathering material. Another source of quartz could be construction sand. Since the study site is located in a densely populated area, the contamination of the samples by anthropogenic material like this sand is very likely.

The lack of olivine in most samples shows the high degree of serpentinization of the rock and the fast degradation of the remaining olivine during weathering.

Serpentine and magnetite are not found in all of the samples; they could already be completely dissolved by weathering. The serpentine is mostly lizardite, which forms in the presence of meteoric-hydrothermal water [35], which fits this setup.

Hematite, goethite (iron oxides), chlorite (iron/aluminum hydroxide) and gibbsite (aluminum hydroxide) are typical weathering products of iron containing minerals. The absence of some of the minerals in some samples indicates different educts, which underlines the theory of the inhomogeneous composition of the Medellín Dunite.

Sample L-07 differs the most from the others. It also contains a small amount of a swelling clay mineral, most likely nontronite. This difference could be due to its remote sampling location separated from the other samples (see Figure 3).

There is no pyroxene found in any of the samples. Pyroxenes are very hard to detect in X-ray diffraction, since their peaks are not very precise but have a range. Their peaks could also be overlapping with the peaks of other minerals.

All samples, also sample L-07, were taken in an anthropogenically highly altered region. Therefore, a contamination, as well as a content of external minerals could not be excluded completely.

## 4. Discussion

The landslide feature map shows only small-sized (max. 15,000 m^2^) and shallow to mid-seated (max. 10 m) landslides. A former, deep-seated landslide could not be excluded, but is nevertheless not very likely. These observations fit into the descriptions of landslides in the eastern slope of Medellín of the past decades [37]. The most devastating landslide in Medellín was the Villatina landslide in 1987, which caused at least 217 casualties [38], according to other sources, even 500 [39]. It was only 1.0–8.0 m deep but had 20,000–40,000 m^3^ of volume [38,39,40]. This indicates that the landslides in the Medellín Dunite are, in fact, mostly shallow to mid-seated ones as registered in the current map. Still, the possibility of a deep-seated landslide is not ruled out completely.

According to the field observations, the soil material at the surface consists almost entirely of the grain sizes of clay and silt. Gravel and sand are absent or only present at some areas in small amounts. This indicates weathering rather than mechanical fragmentation of the rock.

As depicted in Figure 6 and Figure 7, the thickness of the soil below the surface hardly reaches 2 m, except for drilling B1. This was very unexpected and underlines the importance of a detailed and exhaustive investigation of the subsurface in the Medellín Dunite, since this unit shows extreme variations.

### 4.1. Discontinuity Sets

In the geological map, the parallel striking ridges of the dunite are very prominent. According to the general stress field of the region [41], this direction could be a tectonic structure (horst-and-graben structure). The main faults around Medellín (La Acuarela fault, Romeral fault, etc. [41]) strike NNW-SSE with a steep dipping, subvertically, towards WSW. The SW-NE striking structures of the dunite rock cross this fault system vertically with a mediate dipping of around 45° to NW, creating a perpendicular joint system.

At this point, the dipping of the joint systems recorded in the drilling cores could be parallelized with field observations in the outcrops at site (see Figures S1–S3 joint set columns).

Joint set 2 (75–90°) correlates highly with the steep NNW-SSE striking fault system in the region but is highly underrepresented due to its vertical dipping angle. Joint set 3 (25–60°) correlates with the SW-NE striking, vertically crossing structure on the dunite rock surface. Even though the orientation of the joint sets cannot be observed in the drillings, the correlation is possible, since the dipping angles are very distinct (Figures S1–S3). The subhorizontal joint set (joint set 1) [12] also could be observed in the drillings (Figures S1–S3); it is the dominating joint set in all drillings.

### 4.2. Weathering Processes–Serpentinization and “Pseudokarst” Structures

The expected weathering profile of decreasing weathering stages with the depth was not observed in any of the three drillings (see Figures S1–S3 weathering profiles). However, the drillings did confirm the theory of block-in-matrix structures and especially pseudokarst-like cavities in the dunite body, as described by previous studies [12,15]. The core loss in the first 8.9 m of drilling B1 and the high ratio of core loss in all three drillings provide certain evidence of some smaller cavities in the rock mass. Additionally, the presence of large amounts of loose material in deep parts of the drillings indicate a high degree of water circulation within the rock along the pseudokarst structures and other fractures, accumulating the soil material in these cavities and contributing to the high degree of weathering at depth. This phenomenon also creates a block-in-matrix structure in the end.

In all drillings, a serpentinization, especially along fractures and joints, could be observed, besides oxidation processes (brown colored areas). According to literature dealing with weathering of ultramafic rocks [10,12,13,14], this serpentinization is a characteristic weathering process of dunite rock. Normally, serpentinization only takes place in hydrothermal regimes with water temperatures above 100 °C [35]. Antigorite can only be formed above 250 °C [35]. However, since we do observe antigorite and chrysotile in the thin sections, but only lizardite in the X-ray diffraction, lizardite could be the serpentine mineral being formed during weathering processes of dunite rocks, while antigorite and chrysotile were created earlier during ocean floor metamorphosis in a hydrothermal process and are already weathered in the soil samples. This secondary serpentinization could also be the key process of forming the pseudokarst observed. Since pseudokarst is a solution weathering, it is very likely that this process works according to the following serpentinization equation:

2 × Mg_2_SiO_4_ + 3 × H_2_O = Mg_3_Si_2_O_5_(OH)_4_ + Mg(OH)_2_, (1)

Forsterite + Water = Serpentine + Brucite(2)

Brucite has been found as crusts on the surface of the dunite rock in some parts of the Medellín Dunite [12], which underlines the theory of a solution weathering, solving the magnesium olivine forsterite in water circulating in fractures and a precipitation of brucite and the serpentine mineral lizardite in other places within the dunite body. This process would explain the typical karst structures on the surface and the subsurface (dolines, karren, karst caves) as well as the deep weathering of the rock to a depth of at least 60 m [12,15].

The outcomes of the laboratory tests conducted on the samples of the drilling cores show generally no correlation with the depth of the drilling or the drilling locations. This underlines the extreme heterogeneous structure and behavior of the Medellín Dunite. A common weathering profile (decreasing weathering with increasing depth) would show increasing compressive strength, tensile strength and grain sizes with increasing depth [13,14]. Despite the missing correlation between the depth and the values of the rock test results, the values do vary extremely with 10 MPa–132 MPa for uniaxial compressive strength and 4.1 MPa–17.5 MPa for tensile strength. Most samples showing low values were already expected to be weak because of their weathering or serpentinization. However, some samples showed no alteration on the outside, but still had low values in the compressive and tensile strength tests. This shows a varying internal disintegration of the rock’s structure without visible weathering signs even in seemingly intact bedrock to a depth of at least 50 m. Disintegrated rock without oxidation or visible weathering is also observed in the drilling B1 in the homogeneous area 6. These samples seem to be fractured formerly intact rock parts, but can be crushed by hand, indicating an advanced weathering throughout this part of the dunite body without any visible evidence of weathering or tectonic damage already before weathering. However, these parts of the rock show a high degree of serpentinization. In this case, this could be secondary or weathering serpentinization, as mentioned above, which disintegrates the whole rock without oxidation or degradation into clay minerals.

The soil tests also show no correlation of depth with grain size and plasticity of the fine material. Highly plastic soil is distributed throughout the depth of the dunite, cumulating in fractures and pseudokarst caves, which indicates that it originates in weathering of the rock. The loose material is not only oxidized but also shows green colors or no discoloration at all. The green areas could contain lizardite and chlorite, since both minerals are weathering products of the serpentinized dunite rock and show light green colors [35]. Most soil samples provide a high content of fine material, as already observed during field work. This is also visible in the drilling cores since the cores either show intact rock parts or completely disintegrated loose material that does not contain high amounts of gravel. This indicates that the loose material was not formed by tectonic forces (which would have produced gravel and sand alike), but primarily by weathering processes. The plastic behavior of some samples suggests a considerable amount of swelling clay minerals.

Due to the low amount of shear tests possible, the results are only taken into account for understanding coherences. The value gained for the friction angle (24°) and the cohesion (39 kPa) underline some of the results of the Atterberg limits tests, showing a strongly cohesive soil.

### 4.3. Thin Section Analysis and X-ray Diffraction Analysis

The mineralogical composition observed in the thin sections of some rock samples is coherent with previous data [2,3,4,5,6,7,8,9,10,12]. Most parts of the samples are highly serpentinized, antigorite and chrysotile can be distinguished easily, and lizardite could not be found. Therefore, pyroxene, amphibole and olivine (forsterite) are already being transformed to varying degrees, depending on the sample. These differences in the samples regarding the ratio of olivine (forsterite), amphibole and pyroxene and the degree of serpentinization are severe, i.e., amphibole and pyroxene are already dissolved in some samples. The weathering of the material could also be observed, the minerals are mostly transformed into the iron oxide goethite. Even though the presence of olivine in all samples indicates an originally high amount of this mineral in the rock, the amphibole and pyroxene found in most samples indicate that the rock is, in fact, not exactly a dunite, but rather a peridotite. The high degree of serpentinization, however, provides evidence that the unit is mostly made up of serpentinite and not peridotite, harzburgite or dunite.

In the X-ray diffraction, lizardite was found, but no antigorite or chrysotile, indicating a transformation of those two serpentine minerals and the forming of lizardite, possibly by weathering processes. This might underline the theory of a second serpentinization by weathering [10,12,13,14], as explained above. All other minerals observed in the X-ray diffraction were expected after reviewing the thin sections. The quartz in the soil samples could also originate in contamination by construction material in this highly populated area, since it was only found in one thin section but in all the samples taken for the X-ray analyses.

### 4.4. Consequences in the Context of Landslide Hazard Assessment

The consequence of the mineralogy, structure and behavior of the Medellín Dunite discussed above is the landslide probability in the area. Landslides occur predominantly where the rock mass is fragmented by joints and pseudokarst filled with soil [12]. This block-in-matrix structure is created by weathering, whose local intensity (and, therefore, the locally differing amount of soil) is influenced by the mineralogical composition. The thicker and finer the soil (matrix) between the blocks, the higher the probability of a shear surface developing in the soil layer. The probability increases due to water saturation during rainy season or due to anthropogenic reasons, e.g., a leakage in the water pipe system. Since the unit of the Medellín Dunite is so heterogeneous, it is impossible to forecast the location, size, depth and velocity of a landslide event. Therefore, the subsurface has to be investigated and monitored in a high level of detail, especially regarding its structure and composition. To achieve the goal of an improved understanding of the subsurface in the study area, more drillings need to be conducted to obtain the best knowledge possible about the real conditions.

The friction angle of 24° of the fine material derived from the shear test is within the scope of the landslides registered in the past in the Medellín Dunite (SIMMA database). Most of the registered landslides showed an angle of 20–25°. The Villatina landslide, for example, occurred at an angle of 25° at the tear-off edge [40]. However, since the shear test could only be conducted on two samples, further tests need to be done to determine a critical friction angle.

## 5. Conclusions

All mappings and tests conducted indicate a very heterogeneous composition and a diverse behavior of the Medellín Dunite:

- The Medellín Dunite shows variations in its primary mineralogical composition and, therefore, its alteration by serpentinization over short distances and depths.
- Three joint sets could be observed in the drillings that can be correlated with surface structures and known fault and joint systems in the region.
- Both above mentioned predispositions influence the deep weathering of the dunite along fractures. Olivine usually is the most unstable mineral in ultramafic rocks and is the first one to be weathered, as observed in this investigation. When looking at the rock and the soil samples, the olivine is mostly already decomposed or heavily altered by serpentinization and weathering. Therefore, the parts of the Medellín Dunite containing more olivine are weathered faster.
- One weathering characteristic of the Medellín Dunite might be a secondary serpentinization caused by water circulation, primarily creating lizardite and brucite. Indications of this process have been observed in the drilling cores and by comparing the minerals of the rock and the soil samples.
- The pseudokarst observed in the region of the Medellín Dunite and in the current drilling cores might be created by this secondary serpentinization. This phenomenon forms along existing fractures and causes a large number of cavities of different sizes up to 1 m width. The pseudokarst features promote the high water conductivity in the rock mass and, therefore, the high weathering degree in the whole dunite body, reaching a depth of at least 60 m.
- Created by weathering, former landslides and pseudokarst processes, the block-in-matrix structure is the main landslide prone unit at the study site. Its soil content is not as high as expected, but the soil material exists even with increasing depth, is very fine and is therefore suggested to act as a shear surface for landslides, especially when water saturated, even with a low thickness of only a few centimeters.
- A critical friction angle of 20–25°, as most of the former landslides in the SIMMA database, is also to be expected at the study site, as the results from the shear test suggest.
- The transfer of the early warning system created on the basis of the geological findings to other geologically similar study sites is possible. However, a profound investigation of the subsurface in every further area, where the early warning system is to be installed, is indispensable.
- The approach of establishing detailed base data for a Low Cost Early Warning System (LEWS) in deeply weathered ultramafic rock masses is suggested to be applicable to further landslide prone areas.

## Figures and Tables

**Table 1:** Tests performed on the drilling samples, applied standards and number of tests.

Test Performed	Standard	Number of Tests
Compressive Strength	ASTM D 7012-14e1 (2014) [19]	18
Brazilian Test	ASTM D 3967 (2016) [20]	18
Grain Size Analysis	INV E 123 (2013) [21]	7
Atterberg Limits	INV E 125 and INV E 126 (2013) [22,23]	7
Direct Shear Test (CD)	INV E 154 (2013) [24]	1(2)

**Table 2:** Results of the sieving after DIN EN ISO 14688-1 (2020) [30].

Sample	Gravel [%]	Sand [%]	Silt/Clay [%]
A1, 19.40 m	32	21	47
A1, 21.45 m	19	14	67
A1, 25.30 m	15	12	73
A1, 26.00 m	48	15	37
A2, 3.40 m	33	19	48
B1, 2.30 m	2	17	81
B1, 5.25 m	0	7	93

**Table 3:** Results of the Atterberg limits tests, showing sample numbers, plastic and liquid limits, plasticity indices as well as specifications according to the applied standards.

Sample	Plastic Limit [%]	Liquid Limit [%]	Plasticity Index [%]	USCS [32]	DIN 18196 [31]
A1, 19.40 m	26	53	27	SC	TA
A1, 21.45 m	20	31	11	CL	TL
A1, 25.30 m	21	32	11	CL	TL
A1, 26.00 m	27	45	18	GM	GU *
A2, 3.40 m	31	49	18	SM	UM
B1, 2.30 m	72	106	34	MH	UA
B1, 5.25 m	56	77	21	MH	UA

*: the soil contains 15–40 % silt.

**Table 4:** Results of the microscopic analysis of the thin sections, showing the most frequent minerals [34].

	Sample	D-01	D-02	D-03	D-04	D-05	D-06	D-07	D-08.1	D-08.2	D-09	D-10
Mineral												
Olivine		X	X	X	X	X	X	X	X	X	X	X
Serpentine		X	X	X	X	X	X	X	X	X	X	X
Pyroxene			X	X	X	X		X	X	X	X	X
Amphibole				X		X		X		X	X	
Chlorite		X	X	X	X	X	X	X	X	X	X	X
Opaque phase		X	X	X	X	X	X	X	X	X	X	X

**Table 5:** Results of the X-ray diffraction [36].

	Sample	L-01	L-02	L-03	L-04	L-05	L-06	L-07	L-08
Mineral									
Chlorite		X	X	X	X	X	X	X	X
Amphibole (tremolite)		X	X	X	X	X	X	X	X
Serpentine (lizardite)		X	X	X	X		X		X
Gibbsite		X					X		
Magnetite							X		
Quartz		X	X	X	X	X	X	X	X
Goethite		X	X	X	X	X	X		X
Hematite		X	X	X	X	X	X	X	X
Olivine (forsterite)									X

## Supplementary Materials

The following are available online at https://www.mdpi.com/article/10.3390/ijerph182111141/s1, Figure S1: Analysis drilling A1, Figure S2: Analysis drilling A2, Figure S3: Analysis drilling B1.
