# Supplementary material for: Investigation of Critical Geotechnical, Petrological and Mineralogical Parameters for Landslides in Deeply Weathered Dunite Rock (Medellín, Colombia)
Source: Int J Environ Res Public Health. 2021 Oct 23;18(21):11141. doi: 10.3390/ijerph182111141 (PMC8582660; doi:10.3390/ijerph182111141)
Supplement: Supplementary file 1 [file ijerph-18-11141-s001.zip › Figure S2 - Analysis drilling A2.pdf]

Drilling profile | Core loss | Weathering profile | Quality (RQD) | Fracture ratio | Joint set (simplyfied tadpole-depiction) | Alteration type | Homogeneous areas

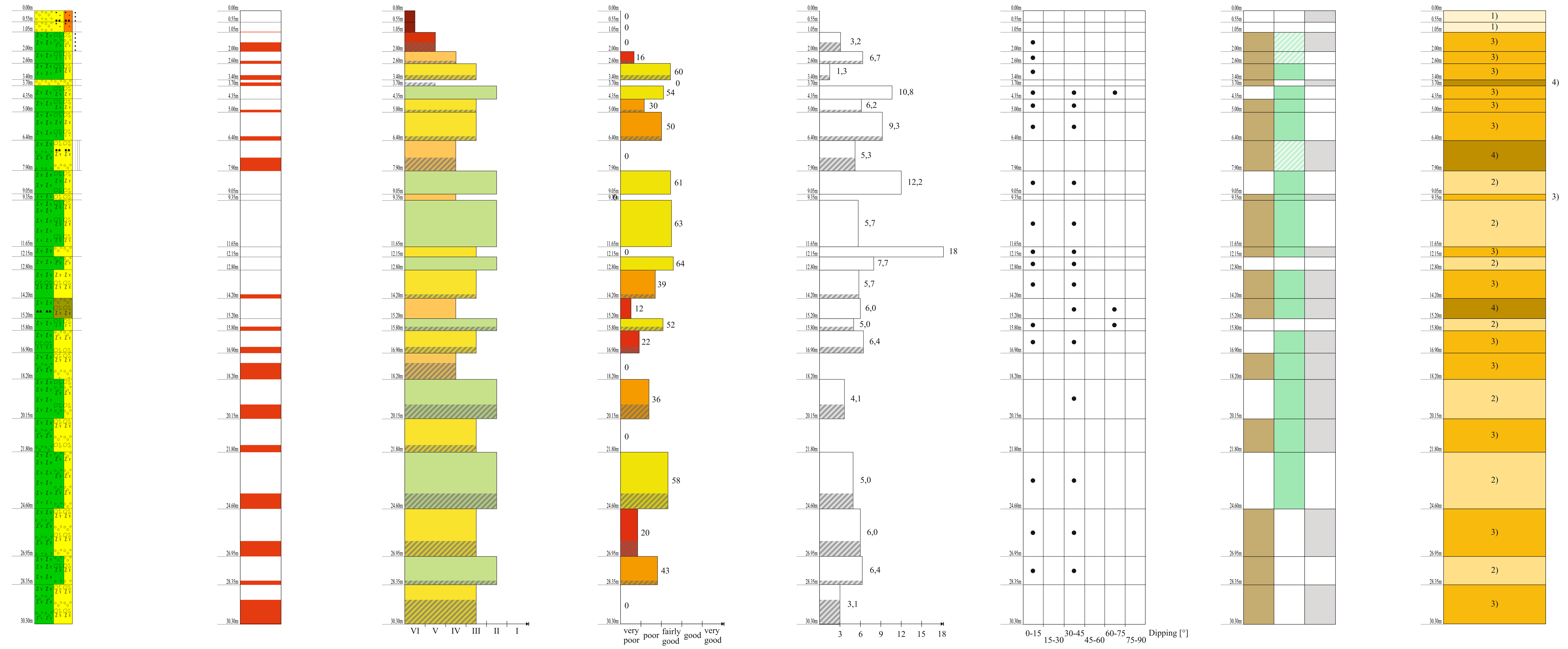

Drilling profile | Core loss | Weathering profile | Quality (RQD) | Fracture ratio | Joint set (simplyfied tadpole-depiction) | Alteration type | Homogeneous areas

Core recover	Core loss
22,86 m	7,44 m
75,45 %	24,55 %

Description of joints (weathering, quantitative)	
I)	fresh
II)	light
III)	moderate
IV)	high
V)	extreme
VI)	soil

RQD	
0 - 25	very poor
25 - 50	poor
50 - 75	fairly good
75 - 90	good
90 - 100	very good

Alteration type	
	brown colored
	serpentinization
	fragmented

Homogeneous areas	
1)	Top soil
2)	Core, lightly weathered
3)	Core, (highly) fractured, lighty weathered
4)	Core, highly fractured, highly weathered
5)	Core, loose, completley fractured, with clay *(here not encountered)*
6)	Core, extremely fragile *(here not encountered)*

Figure S2: Drilling 2020-A2
1.186.291 N; 838.816 E
Bello Oriente, Medellín (Columbia)

Scale 1:100

edited by: Agnes Demharter, Tamara Breuninger
