# Supplementary material for: Investigation of Critical Geotechnical, Petrological and Mineralogical Parameters for Landslides in Deeply Weathered Dunite Rock (Medellín, Colombia)
Source: Int J Environ Res Public Health. 2021 Oct 23;18(21):11141. doi: 10.3390/ijerph182111141 (PMC8582660; doi:10.3390/ijerph182111141)
Supplement: Supplementary file 1 [file ijerph-18-11141-s001.zip › Figure S3 - Analysis drilling B1.pdf]

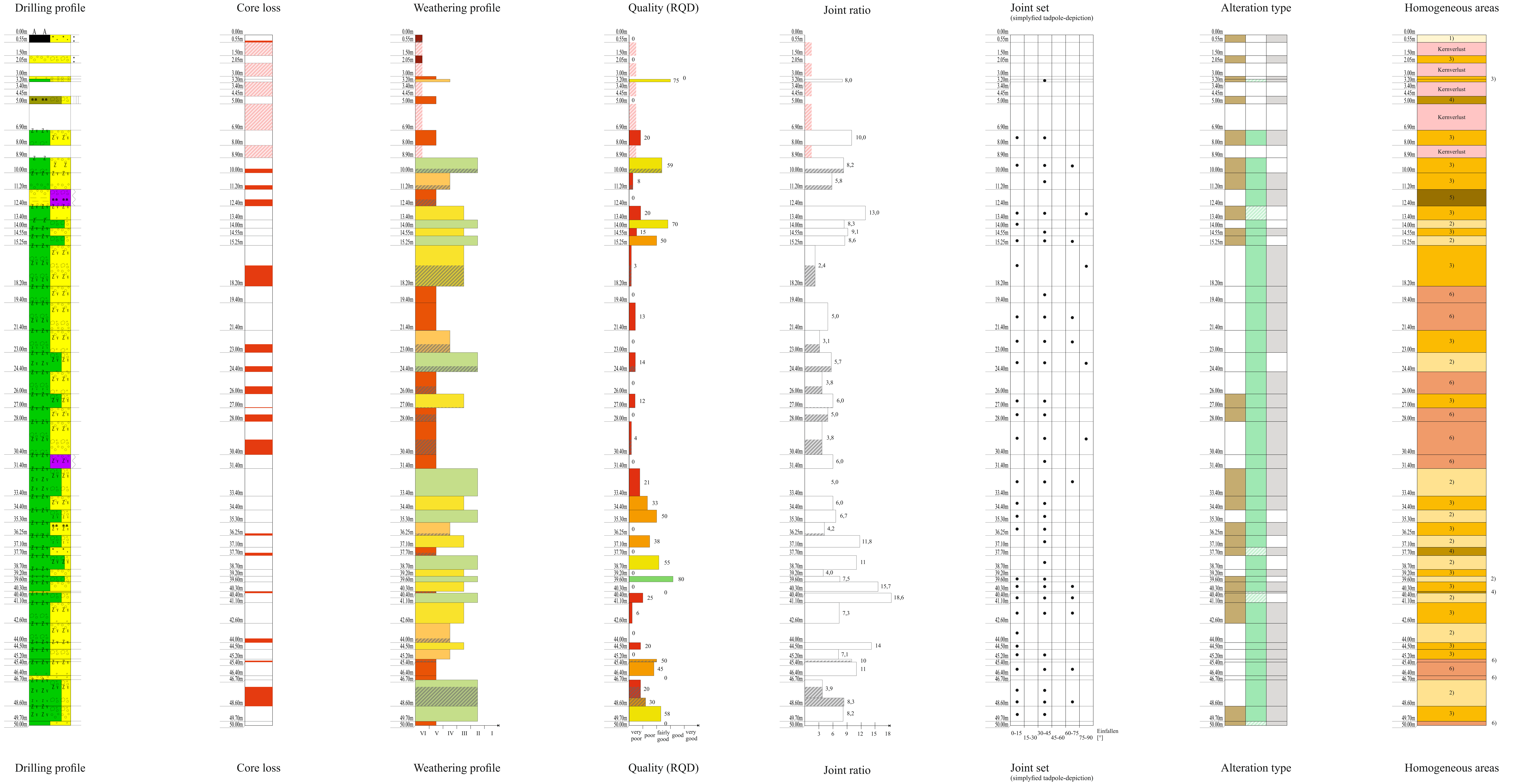

Core loss

Core recover	Core loss
36,11 m	13,89
72,22 %	27,78 %

Weathering profile

Description of joints (weathering, quantitative)	
I)	fresh
II)	light
III)	moderate
IV)	high
V)	extreme
VI)	soil

Quality (RQD)

RQD	
0 - 25	very poor
25 - 50	poor
50 - 75	fairly good
75 - 90	good
90 - 100	very good

Alteration type

Alteration type	
	brown colored
	serpentinized
	fragmented

Homogeneous areas

Homogeneous areas	
1)	Top soil
2)	Core, lightly weathered
3)	Core, (highly) fractured, lighty weathered
4)	Core, highly fractured, highly weathered
5)	Core, loose, completley fractured, with clay
6)	Core, extremely fragile

Figure S3: Drilling 2020-B1
1.186.259 N; 838.622 E
Bello Oriente, Medellín (Columbia)

Scale 1:100

edited by: Agnes Demharter, Tamara Breuninger
